# Exogenous selenium promotes the growth of salt-stressed tomato seedlings by regulating ionic homeostasis, activation energy allocation and CO_2_ assimilation

**DOI:** 10.3389/fpls.2023.1206246

**Published:** 2023-07-04

**Authors:** Wenbo Zhang, Xiaoling He, Xianjun Chen, Hongwei Han, Bingru Shen, Ming Diao, Hui-ying Liu

**Affiliations:** ^1^ Department of Horticulture, Agricultural College, Shihezi University, Shihezi, Xinjiang, China; ^2^ Key Laboratory of Special Fruits and Vegetables Cultivation Physiology and Germplasm Resources Utilization of Xinjiang Production and Construction Crops, Shihezi, Xinjiang, China; ^3^ Institute of Horticultural Crops, Xinjiang Academy of Agricultural Sciences, Urumqi, China

**Keywords:** selenium, tomato, salt stress, ion homeostasis, state transformation, CO_2_ assimilation

## Abstract

This study is aimed at investigating the effects of exogenous selenium (Se) on the ionic equilibrium and micro-domain distribution, state transitions between photosystem I (PSI) and photosystem II (PSII), and the photosynthetic carbon assimilation efficiency of tomato (*Solanum lycopersicon* L.) seedlings under the influence of salt stress. The application of 0.01 mmol•L^-1^ exogenous Se had no significant effects on the selective transport capacity of sodium (Na), potassium (K), calcium (Ca) and magnesium (Mg) from the roots to leaves under salt stress. It, however, significantly hindered the absorption of Na by the root system and leaves, increased the ratios of K/Na, Ca/Na and Mg/Na, and relieved the nonuniformity of micro-domain ionic distribution, thus, mitigating the ionic homeostasis imbalance and ion toxicity induced by salt stress. Additionally, the application of exogenous Se overcame stomatal limitation, regulated the state transitions between PSI and PSII, and enhanced the initial and overall activity of Rubisco as well as the activities of Rubisco activase (RCA) and fructose-1,6-bisphosphatase (FBPase). It also increased the levels of expression of nine relevant genes in Calvin cycle, which subsequently improved the concentration of photosynthetic substrates, balanced the distribution of activation energy between PSI and PSII, promoted the efficiency of CO_2_ carboxylation and carbon assimilation, thereby increasing the photosynthetic efficiency of tomato seedling leaves under salt stress. Hence, the supply of exogenous Se can alleviate the inhibition of salt stress on tomato seedling growth by rebuilding ionic homeostasis and promoting photosynthetic capacity.

## Introduction

Soil salinization is one of the primary factors that inhibit plant growth and constrains yields (Zhu et al., 2016). Salt stress, in turn, subjects the plant to osmotic stress, ion specific toxicity, nutrient deficiencies, and oxidative stress, disrupting many of the physiological and biochemical processes of plants, particularly photosynthesis, and eventually constraining the growth, development, and production of the plant (Hassini et al., 2017; Queiroz et al., 2020). Under salt stress, Na^+^ functions as a toxic ion that competes with K^+^ , Ca^2+^ and Mg^2+^ in the cell, inducing ion toxicity and ionic homeostasis imbalance, which is more likely to cause irreversible damage, besides osmotic stress (Yin et al., 2013; Ren et al., 2020; Hundare et al., 2022). Photosynthesis is closely related to plant growth but is extremely sensitive to adverse stress. Previous studies have revealed that salt stress reduces the photosynthetic assimilation of carbon, transfers feedback of the photosynthetic electron inhibition, and increases the activation energy of photosystem II (PSII), thereby resulting in the accumulation of reactive oxygen species (ROS) and photoinhibition of PSII (Lucini et al., 2016). Therefore, reducing the accumulation of Na^+^, reconstructing ionic homeostasis, and maintaining high photosynthetic performance is a topic that merits urgent study to alleviate the negative effects of salt stress on plants.

Recently, exogenous substances have become an effective way to overcome soil salinization to alleviate the damage of salt stress on crops (Pottosin and Shabala, 2014; Gengmao et al., 2015; Jiang et al., 2016; Shu et al., 2015). Selenium (Se) is an essential trace element for humans and animals (El-Ramady et al., 2014). To date, however, there is no sufficient evidence to prove that Se is an essential micronutrient for plant growth and development. Se can, however not only improve the growth and biological yield of various plants but also significantly improve the Se content in the plant produce, which can meet the needs of human health for Se to some extent (Habibi, 2017; Ashraf et al., 2018; Pecoraro et al., 2022). Although researchers have different ideas about the presence of Se in soil, the forms and concentrations of the application of Se; the absorption, transport and distribution of Se by plants; and the physiological functions of Se in plants have been thoroughly studied. The results confirm Se's participation in various metabolic and physiological activities within plants (Hasanuzzaman and Fujita, 2022). Additionally, many studies have shown that the exogenous Se in low concentrations plays an important role in relieving the damage caused to plants by abiotic stress, such as heavy metals (Kumar et al., 2012; Hu et al., 2014), drought (Hasanuzzaman et al., 2011), high temperature (Djanaguiraman et al., 2010), and salt (Diao et al., 2014; Elkelish et al., 2019). Under salt stress, the application of the optimal concentration of Se can improve the tolerance of plants and promote their growth, promote the absorption of nutrients, adjust osmosis (Xu et al., 2021), and increase oxidation resistance (Habibi, 2017; Elkelish et al., 2019). Elkelish reported that supplementation with Se has a negative effect on the accumulation of Na^+^ in seedlings under salt stress but has positive effects on the Na/K ratio and absorption of N and Ca (Elkelish et al., 2019). The use of Se to support ionic homeostasis, selective absorption and transfer, and micro-domain dispersion under salt stress has, however, received relatively little attention. Additionally, when Se is used under stressful conditions, plant photosynthesis may recover more slowly and be more dependent on reduced levels of active oxygen, reactivated antioxidants, and repaired chloroplasts (Feng et al., 2021).

Yaldiz reported that the application of exogenous Se could alleviate the inhibiting effect of salt stress on sage (*Salvia* sp.) photosynthetic performance and maintain a high rate of photosynthesis (Yaldiz and Camlica, 2021). In our previous work, the concentration of Se that favors seed germination and seedling growth of tomatoes (*Solanum lycopersicon* L.) under salt stress had been confirmed (Han et al., 2010; He et al., 2015). It has also been reported that exogenous Se protected PSII from the damage caused by excess light energy and enhanced the activity of PSII by activating the scavenging activity of ROS and non-photochemical quenching (NPQ) mechanism in the chloroplast (Diao et al., 2014). However, the ability of Se to adjust the state transitions between photosystem I (PSI) and II and carbon assimilation efficiency is still unclear. Therefore, this study aimed to revealed the mechanism by which exogenous Se improves the salt tolerance of tomato seedlings based on the ionic equilibrium micro-domain distribution, state transitions between PSI and II, and efficiency of carbon assimilation.

## Materials and methods

### Plant materials and treatment conditions

A tomato hydroponic experiment was conducted in a greenhouse. The test material was the tomato variety 'Shuangfeng 87-5'. The seeds were germinated in a plug tray with a mixture of peat and vermiculite (1:1, v/v). When the two true leaves were fully expanded, tomato plants of uniform shape were selected and transplanted into a 12 L bucket containing Hoagland’s nutrient solution, prepared by adding 10 L of deionized water to the bucket, and the pH was maintained at 6.2 by adding sulfuric acid (H_2_SO_4_) or potassium hydroxide (KOH). After the seedlings had grown four true leaves, NaCl and sodium selenite (Na_2_SeO_3_) were added to the nutrient solution, and the treatment of salt and Se began. Four groups were established for the experiment: (a) control group: no Se or NaCl; (b) Se: 0.01 mmol•L^-1^ Na_2_SeO_3_; (c) NaCl: 100 mmol•L^-1^ NaCl; and (d) NaCl + Se: 0.01 mmol•L^-1^ Na_2_SeO_3_ + 100 mmol•L^-1^ NaCl. The concentrations and application methods of Na_2_SeO_3_ and NaCl used herein were based on the results of our prior study (He et al., 2015). The experiment utilized a randomized block design with three replicates per treatment. The time of illumination was approximately 14 h; the temperature was recorded as 24 ~ 30 °C in the daytime and 17 ~ 20 °C at night, and the nutrient solution was changed every three days.

### Growth measurements

The growth indexes of plants were measured according to the method of Chen et al. (2010) and Chen et al. (2021). Following 9 d of treatments, the plant height was measured with a ruler, and the stem diameter was measured using a Vernier caliper. Using the base of the stem as the point of reference, the tomato seedlings were divided into aboveground and belowground parts, and tap water was used to rinse them 2-3 times. Distilled water was then used to rinse them twice. The fresh weight was measured after the absorption of moisture using an absorptive paper. The seeding sample was quenched at 105 °C for 15 min, heated at 75 °C until the sample had reached a constant weight, and weighed as the dry mass to constant weight in the unit of grams.

### Determination of the contents of elements

Following 9 days of treatments, dry samples of the leaves and root system were ground, and 0.2 g of powder was added to a digestion tube. 8 mL of nitric acid (HNO_3_) and 4 mL of hydrogen peroxide (H_2_O_2_) were added, and the samples were digested by CEM (CEM Corporation, Matthews, NC, USA) microwave digestion and extraction instrument. After digestion, the acid was heated by an electric heating plate until there was only one drop of acid left in the digestion tube. This drop of acid was then diluted to 50 mL. The contents of K, Ca, Na, Mg and Se in the leaves and root system of the tomato seedling systems were determined using inductively coupled plasma atomic emission spectrometry (ICP-AES, Thermo Scientific ICAP 6000 Series, Boston, MA, USA). The content ratios of mineral elements (Na/K, Na/Ca, and Na/Mg) were also determined. To evaluate the ability of tomato seedling root system to select and transfer mineral elements to leaves, the element selection transport coefficient [S_Na, X_ = Na (leaf/root) /X(leaf/root), wherein X stands for K, Mg, or content of Ca] was calculated as described by (Zhou et al. (2019).

### X-ray micro-analysis of Na^+^, Cl^-^, K^+^, Ca^2+^, and Mg^2+^ in the leaves and root system

**Figure 1:**
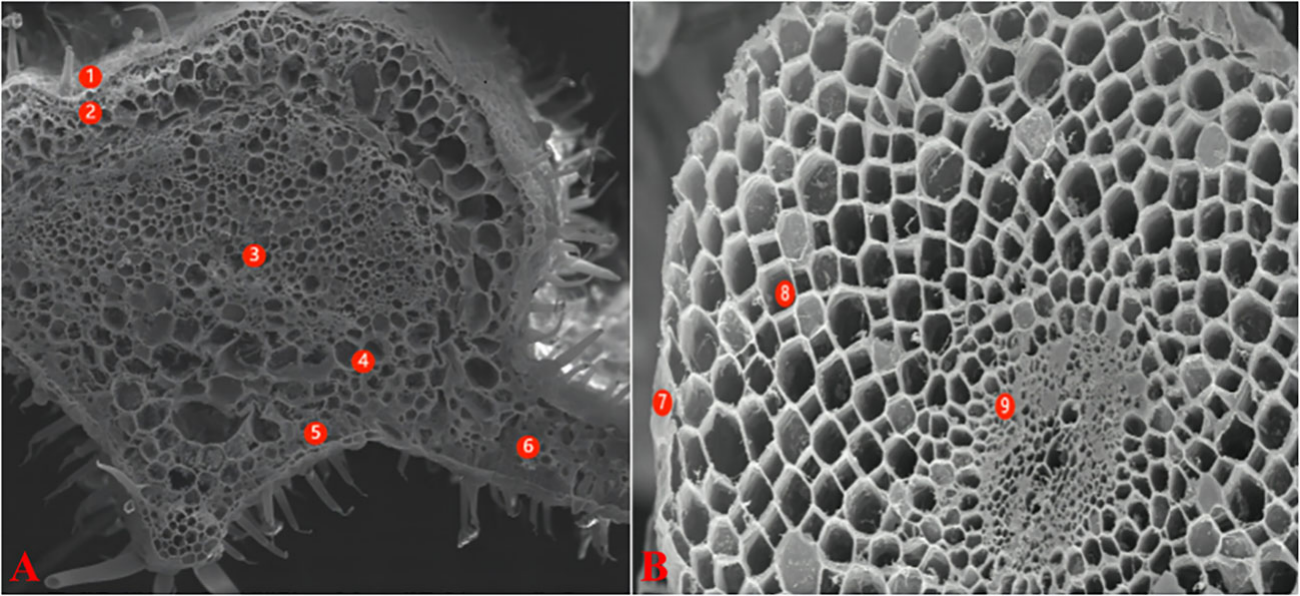
Electron-microscope scanning image of freeze-vacuum-dried cross-section of different organs tomato. **(A)**: leaf; **(B)**: root. Leaf: 1-Epidermis; 2-Parenchyma; 3-Xylem; 4-Phlocm; 5-Spongy tissue; 6-Palisade tissue Root: 7-Epidermis; 8-Cortex; 9-Stele.

For X-ray micro-analysis, the samples were processed as described by Lei et al. (2014). The base part of the main veins of leaves was cut with a sharp blade as the leaf samples. The leaves were wrapped in tin foil, flash frozen in liquid nitrogen, and vacuum dried. The samples were examined with a field emission scanning electron microscope (FE-SEM) LEO1530 (Zeiss, Oberkochen, Germany). The maximum acceleration voltage was 15.0 keV; the sample inclination angle was 0°, and the angle between the sample and the probe was 35°. With the standard sample program, the element species represented by each peak were determined. The percentages of K^+^, Na^+^, Mg^2+^, Cl^-^ and Ca^2+^ in the total weight of measured ions in the cells were calculated automatically. According to the anatomical structure of tomato leaves, the micro-areas analyzed included the epidermis, xylem, phloem, parenchyma, palisade tissues, and spongy tissue cells (**Figure 1A**). The root section was removed 3-4 cm from the root tip with a double-sided blade, and then treated in a manner similar to the leaves. The analyzed micro-areas included the epidermis, cortex, and cells in the middle column (**Figure 1B**).

### Determination of the gas exchange parameters

After 9 days of treatments, the net photosynthesis rate (Pn), stomatal conductance (Gs), transpiration rate (Tr), and intercellular CO_2_ concentration (Ci) of the third functional leaf for tomato plants from the top of the growing point was measured using a portable photosynthesis system (CIRAS-2; PP Systems, Amesbury, MA, USA). A photo-response curve was established, and the relative humidity was established as 70%. The CO_2_ was from the atmosphere, and the instrument light intensity was successively set to 1400, 1200, 1000, 800, 600, 400, 300, 200, 100, 50, 0 μmol•m^-2^•s^-1^. The classical Farquhar model was used to fit the light response curve. The dark respiration rate (*Rd*) and apparent quantum yield by photosynthesis (*AQY*) were calculated in accordance with the theoretical formula of the model. To measure and establish the response curve of CO_2_-photosynthesis (A/Ci curve), the light intensity was established as 800 μmol•m^-2^•s ^-1^; the leaf temperature was 25±1.5 °C, and the air humidity was 70%. The CO_2_ concentration of the leaf chamber of the instrument was successively set to 50、100、200、300、500、700、900、1200 and 1500 μmol•mol^-1^. The maximum carboxylation rate of Rubisco (*Vc*,*max*, μmol•m^-2^•s^-1^) and the maximum electron transport rate (*Jmax*, μmol•m^-2^•s^-1^) were calculated as described by Ethier and Livingston (2004).

### Determination of the excitation energy allocation

As described by Zhou et al. (2019), a portable fluorometer (PAM-2100, Walz, Germany) was used to measure and calculate the chlorophyll fluorescence parameters including steady-state chlorophyll fluorescence (Fs), initial fluorescence (Fo′), maximum fluorescence yield (Fm′), and Fv′=Fm′-Fo′. The distribution coefficients of the activation energy in two photosystems were calculated as follows: *α*=f/(1+f), *β*=1/(1+f), f=(Fm′-Fs)/(Fm′-Fo′), and the imbalance of activation energy distributed between PSI and II was represented by *β/α*-1. The fraction of excitation energy absorbed in PSII antennae utilized for photosynthetic electron transport (*P*) , dissipated *via* thermal energy in the antenna ( *D*), and the fraction of excess excitation energy that is neither dissipated in the PSII antennae nor utilized for photochemistry (*Ex*), were calculated using formulae reported previously by Li et al. (2004).

### Assay of the activities of Rubisco, RCA, and FBPase

A total of 0.3 g tomato leaves samples were snap-frozen in liquid nitrogen, and 3 mL of precooled Tris-HCl with a concentration of 50 mmol•L^-1^ extract was added, including 1 mmol•L^-1^ EDTA and 1 mmol• L^-1^ magnesium chloride (MgCl_2_). A volume of 12.5% glycerol (v/v), 10% soluble polyvinylpyrrolidone-40 (PVP-40), and 10 mmol•L^-1^ β-mercaptoethanol was added before use. The mixture was homogenized and then centrifuged at 15000 × g for 15 min. The operation was carried out at a temperature between 0 and 4 °C, and the supernatant was the crude extract of Rubisco. According to (Jin et al., 2006), the initial and total activities of Rubisco were assessed. The manufacturer's instructions for the plant RCA and FBPase ELISA kits (TIANDZ, China) were followed in determining the activity of Rubisco activase (RCA) and fructose-1,6-bisphosphatase (FBPase).

### Total RNA extraction and analysis of gene expression

The total RNA was extracted using TRIzol (Sangon, China). cDNA synthesis was performed using a reverse transcription kit (TIANDZ). Reverse transcription PCR (RT-PCR) was performed using nine specific gene primers of Calvin cycle genes as shown in **Table 1**. The iCycler iQ Multicolor Real-Time PCR Detection System (Bio-Rad, Hercules, CA, USA) and SYBRGreen RT PCR fluorescent dye kit were used to detect the products of RT-PCR. The 20 μL reaction system contained 10 μL SYBR Green QPK-201, 0.8 μL primers for the sense and antisense ends, 1 μL cDNA template, and 7.4 μL ddH_2_O. The PCR cycle was performed as follows: initial denaturation at 95 °C for 30 s, denaturation at 95 °C for 5 s, annealing at 58 °C for 45 s, and extension at 72 °C for 15 s. In the 56 °C step, data were collected, and 40 cycles were repeated. The fluorescence value of *actin*, which is primarily the housekeeping gene of tomatoes, was used as the internal standard for calculation, and the relative gene expression was calculated using the 2^-ΔΔCt^ method (Song et al., 2014).

**Table 1:** Primers used for real time RT-PCR assays.

Gene	Forward Primer	Reverse Primer
*Actin*(SLU60478)	5’-TGGTCGGAATGGGACAGAAG-3'	5’-CTCAGTCAGGAGAACAGGGT-3'
*RbcL* (XM_012015910.1)	5'-TTTCCAAGGTCCGCCTCA-3'	5'-CCACCGCGAAGATATTCATA-3'
*RbcS* (XM_015214078.1)	5'-TGTGGAAGTTGCCTATGTTTGG-3'	5'-GCACTTGACGCACATTGTCG-3'
*RCA*(XM_016041479.1)	5'-TACACCGTCAACAACCAG-3'	5'-GGATAAGAGGAGCATACAAT-3'
*SBPase* (XM_006355592.2)	5'-ATGGGAAACAATCCGTCCTT-3'	5'-CTCAAACAGCAGCACCAACT-3'
*PGK*(NM_001318543.1)	5'-AGCGGTTGAGAAAGTTGGAG-3'	5'-GCTATGACACCAGGGAGCAC-3'
*PRK*(XM_006352860.2)	5'-TGTTCTTACCCTGGCATCAA-3'	5'-TGCTCAAATGGCTCTCCAC-3'
*FBPase* (XM_015232533.1)	5'-TACAGCCCGAATGATGAGTG-3'	5’-GAAGGTTTGTCCCTGGTTGA-3'
*TK* (XM_015200072.1)	5'-CATTCTGATGTTCCGTCCAG-3'	5'-CCGAGAGAGGGCAAGGATT-3'
*GAPDH* (NM_001279325.2)	5'-CTGACAAGGACAAGGCTGCT-3'	5-CCTCAACAATGCCAAACCTA-3'

actin, Actin gene; RbcL, Rubisco large subunit gene; RbcS, Rubisco small subunit gene; RCA, Rubisco activase gene; SBPase,sedoheptulose-1,7-bisphosphatase gene; PGK, Phosphoglycerate kinase gene; PRK, Phosphoribulokinase gene; FBPase, Furetose-l,6-bisphosPhate phosphatase gene; TK, Transketolase gene; GAPDH, Glyceraldehyde 3-phosphate dehydrogenase gene.

### Statistical analysis

SPSS 19.0 (IBM, Inc., Armonk, NY, USA) was used for a one-way analysis of variance (ANOVA), and Duncan’s new complex range method was adopted to compare the differences among different treatments. *P < 0.05* indicates a significant difference. Values in the chart denote mean ± standard deviation (SD).

## Results

### Plant growth

The growth of tomato seedlings is inhibited by the presence of 100 mmol•L^-1^ NaCl, as shown in **Table 2**, as evidenced by the fact that plant height, stem diameter, dry weight, and fresh weight of the above-ground and below-ground parts of tomato seedlings decreased significantly in comparison to the control group. The stem diameter, dry weight, and fresh weight of the above-ground and below-ground sections of the tomato seedlings, however, were positively impacted by the application of exogenous Se, which counteracted the negative effects of NaCl stress. However, exogenous Se administration exhibited no discernible impact on the plant height, stem diameter, dry weight, and fresh weight of the above-ground and below-ground sections of tomato seedlings, in the absence of salt stress.

**Table 2:** Effects of exogenous Se on plant height, stem diameter, fresh weight (FW) of shoot and root, dry weight (DW) of shoot and root in tomato seedlings under NaCl stress.

Treatment	Plant Height (cm)	Stem diameter (cm)	Shoot FW (mg kg^−1^ FW)	Root FW (mg kg^−1^ FW)	Shoot DW (mg kg^−1^ DW)	Root DW (mg kg^−1^ DW)
Control	30.70±0.95ab	0.77±0.02a	42.60±2.42a	16.87±0.42a	4.23±0.23a	0.99±0.04ab
Se	31.90±1.76a	0.77±0.03a	41.29±0.48a	16.69±0.77a	4.32±0.07a	1.07±0.11a
NaCl	27.27±0.29c	0.61±0.01c	22.85±1.43c	13.45±0.67c	2.31±0.15c	0.76±0.05c
NaCl + Se	29.13±0.68bc	0.65±0.01b	28.73±0.92b	15.00±0.71b	2.85±0.12b	0.88±0.04b

The mean values (±SD) in each column followed by different letters indicate significant differences at 0.05 level among the treatments according to the Tukey’s multiple range test. Control: no Se and no NaCl; Se: 0.01 mM Na_2_SeO_3_; NaCl: 100 mM NaCl; and NaCl+Se: 0.01 mM Na_2_SeO_3_+100 mM NaCl.

### Content of the elements in leaves and root system

**Figure 2:**
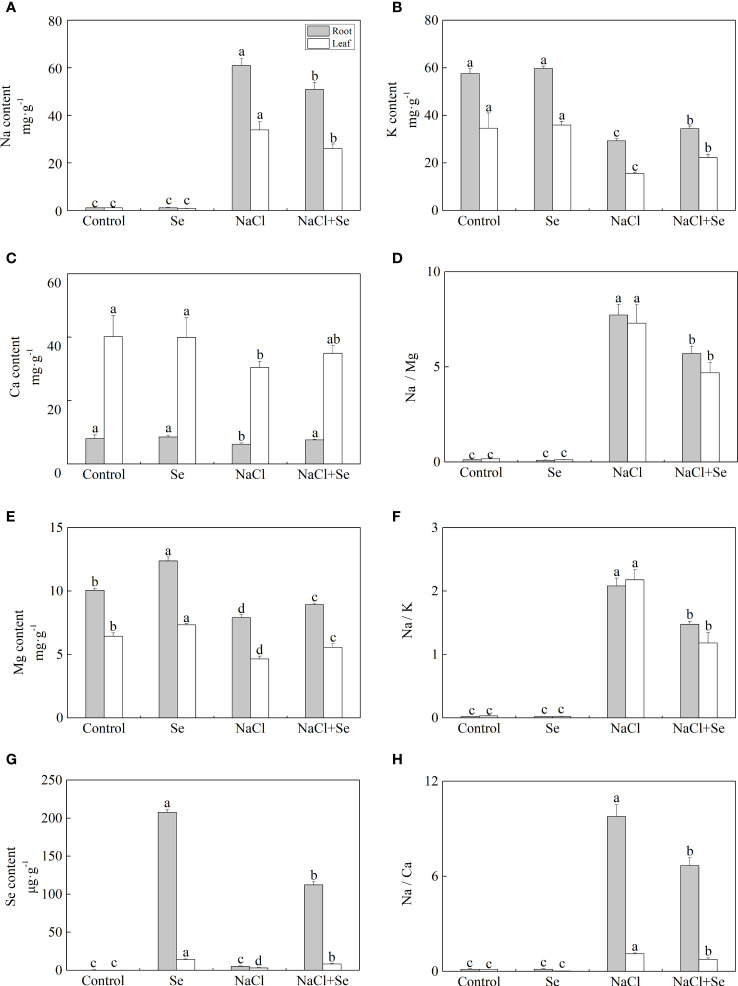
Effects of exogenous Se on the element contents of Na **(A)**, K **(B)**, Ca **(C)**, Mg **(E)** and Se **(G)** , and the ratios of Na/Mg **(D)**, Na/K **(F)**, and Na/Ca **(H)** in leaves and roots of tomato seedlings under NaCl stress. All the measurements were made on leaves at 9 d after treatment. Error bars represent SD (n = 3). Different letters indicate the significance differences among the treatments (*P* < 0.05). Control: no Se and no NaCl; Se: 0.01 mM Na_2_SeO_3_; NaCl: 100 mM NaCl; and NaCl+Se: 0.01 mM Na_2_SeO_3_+100 mM NaCl.

**Figures 2A–C, E, G** shows that compared with the control group, the application of exogenous Se alone had no significant effects on the contents of Na, K, and Ca in the leaves and root system of tomato seedlings, however, it led to an increase in the content of Mg and Se. Under NaCl stress, the contents of Na in the leaves and root system of tomato seedlings increased by 2,936.5% and 5,338.8%, while the contents of K, Ca and Mg decreased by 55.11% and 24.24%, 27.70%, and 49.15%, and 22.23% and 21.45%, respectively. Compared with the NaCl group, the content of Na in the leaves and root system of tomato seedlings after the application of Se was reduced by 23.08% and 16.47% under salt stress, respectively. The contents of K, Ca, and Mg increased by 42.86% and 14.73%, 19.49%, 14.47%, and 22.16% and 13.05% under NaCl stress with Se application, respectively. The application of Se also improved the content of Se in the leaves and root systems of tomato seedlings, while it was significantly lower than that after Se application alone (**Figure 2G**). Additionally, salt stress significantly increased the ratios of Na/K, Na/Ca, and Na/Mg of the leaves and root system (**Figures 2D, F, H**). Compared with the NaCl group, the application of Se counteracted the negative impact of NaCl stress on the ratios of Na/K, Na/Ca, and Na/Mg in the leaves and root system.

### Element transportation capacity from the roots to leaves

**Figure 3:**
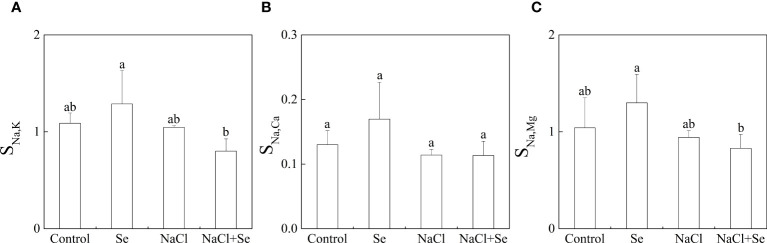
Effects of exogenous Se on the element selection transport coefficients S_Na,k_ **(A)**, S_Na,Ca_ **(B)** and S_Na,Mg_ **(C)** in tomato seedlings under NaCl stress. All the measurements were made on leaves at 9 d after respective treatment. Error bars represent SD (n = 3). Different letters indicate the significance differences among the treatments (*P* < 0.05). Control: no Se and no NaCl; Se: 0.01 mM Na_2_SeO_3_; NaCl: 100 mM NaCl; and NaCl+Se: 0.01 mM Na_2_SeO_3_+100 mM NaCl.

As shown in **Figure 3**, salt stress had no significant effects on S_Na,K_, S_Na,Mg_ and S_Na,Ca_; S_Na,K_, S_Na,Mg_ and S_Na,Ca_ of the Se group, and the NaCl + Se group differed only slightly from the control and NaCl groups, respectively, indicating that application of exogenous Se had no significant effects on the selective absorption and transport capacity of Na, K, Mg and Ca from the root system to leaves under salt stress.

### Ion micro-distribution in the leaves and root system

**Table 3** provides the relative contents of Na^+^, Cl^-^, K^+^, Ca^2+^, and Mg^2+^ in the epidermis, phloem, xylem, palisade tissues, and spongy tissue cells of the tomato seedling leaves under different treatments. Na^+^ was not detected in any tissue cells of the leaves of the control group, while the application of exogenous Se led to significantly enhanced relative K^+^ ion content in the epidermis, xylem, phloem, and spongy tissues, Ca^2+^ in the epidermis and phloem, and Mg^2+^ in the epidermis and spongy tissues. There was also a significant reduction in the relative content of Ca^2+^ in the xylem. Na^+^ also was not detected in any leaf tissues in the Se group. NaCl stress led to increased relative contents of Na^+^ and Cl^-^ and significantly reduced the relative contents of K^+^ and Ca^2+^ in all the leaf tissues. Mg^2+^ was not detected in the epidermis, xylem, or phloem. In particular, Na^+^ and Cl^-^ in the xylem and phloem increased substantially, whereas Cl^-^ increased by 1,201.68% and 1,069.14%, respectively. The amount of K^+^ in the phloem and palisade tissues decreased significantly; by an amount of 95.85% and 93.29%, respectively. The amount of Ca^2+^ in the xylem and palisade tissues demonstrate a relatively large decrease amounting to 81.79% and 85.56%, respectively. Compared with the NaC1 treatment, the NaC1+Se treatment improved the relative contents of K^+^ in all the leaf tissues except for the spongy tissues. It increased the concentration of Ca^2+^ in the epidermis, palisade tissues, and spongy tissues; that of Mg^2+^ in the epidermis and palisade tissues; and of Cl^-^ in the phloem and palisade tissues. The relative contents of K^+^ in the epidermis, xylem, and phloem manifested substantial increases of 289.47%, 283.52%, and 273.44%, respectively. The relative contents of Ca^2+^ in the palisade tissues had a maximum increase of 288.04 %. However, the relative contents of Na^+^ in all the leaf tissues except spongy tissues, that of Cl^-^ in the epidermis, and of Mg^2+^ in the xylem and spongy tissues manifested a significant decrease. Additionally, Mg^2+^ was not detected in the epidermis, xylem or phloem.

**Table 3:** Effects of exogenous Se on the relative contents of ions in leaf tissues of tomato seedlings under NaCl stress.

Leaf tissue	Treatment	Relative content of ions (%)				
		K^+^	Ca^2+^	Mg^2+^	Cl^-^	Na^+^
Epidermis	Control Se NaCl NaCl+Se	8.11±0.73b 11.46±0.67a 0.76±0.13d 2.96±0.58c	3.42±0.27b 5.80±0.48a 1.09±0.07d 2.34±0.11c	1.29±0.17a 1.04±0.12b - 0.30±0.04c	2.37±0.38c 2.38±0.43c 15.20±0.67a 11.67±0.71b	- - 5.42±0.54a 3.96±0.78b
Xylem	Control Se NaCl NaCl+Se	14.70±1.24b 30.04±1.84a 1.76±0.43d 6.75±0.84c	18.56±1.25a 8.23±1.24b 3.38±0.44c 2.17±0.62c	0.60±0.07a 0.68±0.15a - -	3.57±0.41c 3.65±0.51c 49.47±5.92a 24.11±4.48b	- - 13.61±2.18a 6.86±0.25b
Phloem	Control Se NaCl NaCl+Se	30.84±1.11b 35.03±1.61a 1.28±0.23d 4.78±0.22c	2.74±0.23b 4.39±0.54a 0.64±0.04c 0.82±0.05c	0.69±0.15a 0.62±0.07a - -	1.75±0.19c 3.41±0.19c 20.46±1.21b 30.74±2.01a	- - 15.82±1.60a 9.93±1.18b
Palisade tissue	Control Se NaCl NaCl+Se	15.80±1.59a 15.89±1.29a 1.06±0.08c 3.97±0.51b	6.37±0.78a 6.16±0.72a 0.92±0.05c 3.57±0.25b	0.83±0.07a 0.86±0.04a 0.36±0.03c 0.58±0.07b	3.72±0.33c 2.93±0.26c 7.67±1.37b 15.73±0.53a	- - 6.41±0.90a 4.50±0.31b
Spongy tissue	Control Se NaCl NaCl+Se	3.85±0.02b 5.30±0.53a 1.58±0.35c 1.29±0.30c	9.73±0.58a 9.38±0.59a 2.60±0.52c 4.27±1.24b	0.57±0.07b 0.75±0.11a 0.60±0.06b 0.33±0.07c	3.35±0.27c 3.04±0.52c 9.97±1.17a 6.49±0.40b	- - 5.66±0.51a 5.99±0.23a

All the measurements were made on leaves at 9 d after treatment. Error bars represent SD (n = at least 3). Different letters indicate the significance differences among the treatments (P < 0.05). Control: no Se and no NaCl; Se: 0.01 mM Na_2_SeO_3_; NaCl: 100 mM NaCl; and NaCl+Se: 0.01 mM Na_2_SeO_3_+100 mM NaCl.

As shown in **Table 4**, in the absence of NaCl stress (control group), Na^+^ and Mg^2+^ were not detected in the epidermis, cortex, and mid-column. There was a relatively large content of K^+^ in the epidermis, cortex and mid-column. The application of Se alone improved the relative contents of K^+^ in the epidermis and cortex and of Mg^2+^ in the epidermis and mid-column of the root system of tomato seedlings. However, there were no significant differences in the relative contents of Ca^2+^ and Cl^-^ in the different tissues of the root system. NaCl stress improved the relative contents of Na^+^ and Cl^-^ in different tissues of the root system, while the relative contents of Na^+^ and Cl^-^ increased most significantly in the cortex by 1,908% and 519%, respectively. There was a decrease in the relative contents of Mg^2+^ in the epidermis and cortex and that of K^+^ in the epidermis and mid-column, while the relative contents of Ca^2+^ in all the tissues exhibited negligible changes. Under NaCl stress, the application of exogenous Se led to a significant reduction in the relative contents of Na^+^ in different tissues of the root system, of Cl^-^ in the epidermis and cortex, and of K^+^ in the cortex. The relative contents of Na^+^ in the mid-column were reduced to a level below LOD. The relative contents of Ca^2+^ in different tissues and the relative contents of Mg^2+^ in the epidermis and cortex of the root system under NaCl +Se treatment also significantly increased.

**Table 4:** Effects of exogenous Se on the relative contents of ions in root tissues of tomato seedlings under NaCl stress.

Root tissue	Treatment	Relative content of ions (%)				
		K^+^	Ca^2+^	Mg^2+^	Cl^-^	Na^+^
Epidermis	Control Se NaCl NaCl+Se	8.64±0.41b 10.21±0.99a 0.59±0.06c 0.90±0.13c	0.95±0.05bc 1.27±0.09b 0.61±0.13c 1.93±0.64a	0.93±0.04b 1.05±0.09a 0.56±0.06c 0.86±0.03b	1.77±0.02c 1.46±0.24c 8.26±0.92a 3.39±0.59b	- 0.45±0.06c 4.24±0.06a 2.87±0.34b
Cortex	Control Se NaCl NaCl+Se	1.48±0.70c 17.77±0.27a 3.34±0.82b 1.58±0.35c	0.90±0.06b 0.93±0.07b 0.69±0.03b 4.38±0.36a	0.98±0.08a 0.93±0.04ab 0.65±0.03c 0.82±0.11b	3.70±1.76bc 2.73±0.28c 11.02±1.38a 5.50±0.35b	- - 6.01±0.09a 3.91±0.25b
Stele	Control Se NaCl NaCl+Se	39.47±1.19a 35.14±1.00b 6.29±0.71c 4.91±0.44c	1.50±0.39bc 1.10±0.19c 1.10±0.19c 7.02±0.17a	- 0.83±0.02 - -	3.86±1.24c 3.78±0.17c 7.48±1.36b 13.48±0.78a	- - 4.56±0.71 -

Error bars represent SD (n = at least 3). Different letters indicate the significance differences among the treatments (P < 0.05). Control: no Se and no NaCl; Se: 0.01 mM Na_2_SeO_3_; NaCl: 100 mM NaCl; and NaCl+Se: 0.01 mM Na_2_SeO_3_+100 mM NaCl.

### Gas exchange parameters

**Figure 4:**
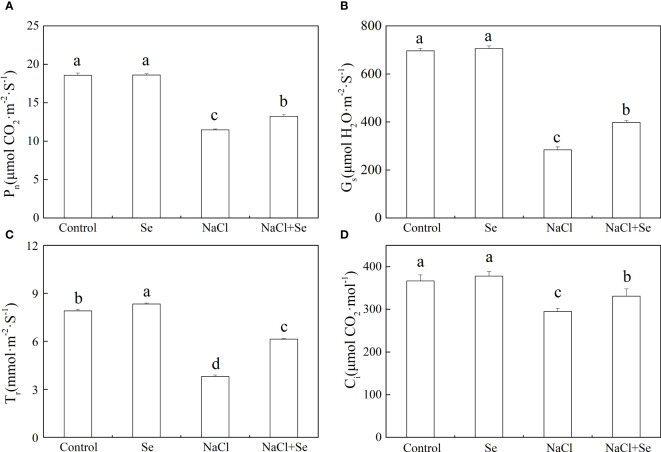
Effects of Se application on the net photosynthetic rate (Pn) **(A)**, stomatal conductance (Gs)**(B)**, intercellular CO_2_ concentration (Ci) **(C)**, and transpiration rate (Tr) **(D)** in leaves of tomato seedlings under salt stress. All the measurements were made on leaves at 9 d after treatment. Error bars represent SD (n = 3). Means denoted by the same letter did not significantly differ at *P* < 0.05 according to Tukey’s test. Control: no Se and no NaCl; Se: 0.01 mM Na_2_SeO_3_; NaCl: 100 mM NaCl; and NaCl+Se: 0.01 mM Na_2_SeO_3_+100 mM NaCl.

As shown in **Figure 4**, the application of Se had no significant effects on the values of Pn, Gs, and Ci but led to an increase in the Tr. Compared with the control group, salt stress significantly decreased the values of Pn, Gs, Ci and Tr. The application of exogenous Se counteracted the effects of salt stress on the values of Pn, Gs, Ci and Tr in the tomato leaves, which in comparison with the NaCl group increased by 15.41%, 40.14%, 12.33%, and 61.40%, respectively.

### The allocation of the fraction of excitation energy

**Figure 5:**
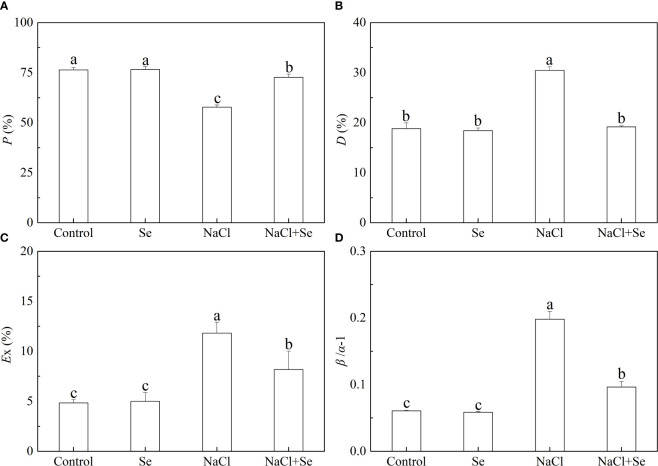
Effects of Se application on the fraction of photon energy absorbed in PSII antennae utilized for photosynthetic electron transport *(P)* **(A)**, the fraction of photon energy absorbed in PSII antennae and dissipated via thermal energy in the antenna *(D)* **(B)**, the estimate of the fraction of excess excitation energy that is neither dissipated in the PSII antennae nor utilized for photochemistry *(Ex)* **(C)** and the relative deviation from full balance *(β/α–1)* **(D)** between PSI and PSII in leaves of salt-stressed tomato seedlings. All the measurements were made on leaves at 9 d after treatment. Error bars represent SD (n = 3). Different letters indicate the significance differences among the treatments *(P < 0.05)*. Control: no Se and no NaCl; Se: 0.01 mM Na_2_SeO_3_; NaCl: 100 mM NaCl; and NaCl+Se: 0.01 mM Na_2_SeO_3_+100 mM NaCl.

As shown in **Figure 5**, the *P* values of tomato leaves under salt stress decreased by 24.39%, and *β/α-1*, *D* and *Ex* of the tomato leaves increased by 226.36%, 61.93%, and 144.31%, respectively, compared with the control group, indicating that the application of exogenous Se led to an increase in the *P* value and significantly reduced the values of *D*, *Ex*, and *β/α-1* under salt stress. These parameters did not difference significantly between the control group and the Se group.

### *Rd, AQY, Vc max*, and *Jmax*

**Figure 6:**
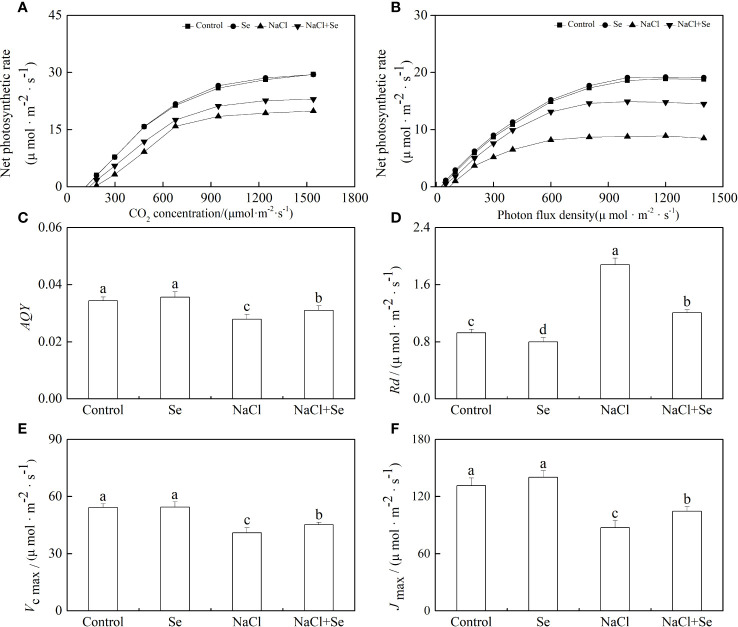
Effects of Se application on the CO_2_-photosynthesis response curves **(A)** and photo-response curves **(B)**, apparent photosynthetic quantum efficiency *(AQY)* **(C)** and the dark respiration rate under light *(Rd)* **(D)**, the maximum Rubisco carboxylation rate *(Vc,max)* **(E)** and maximum ribulose-1,5-bisphosphate (RuBP) regeneration rates *(Jmax)* **(F)** in leaves of tomato seedlings under salt stress. All the measurements were made on leaves at 9 d after respective treatment. Data are means of four biological replicates (±SD). Means denoted by the same letter did not significantly differ at P < 0.05 according to Tukey’s test.

As shown in **Figures 6C**, **E**, NaCl stress led to a decrease of 18.89% in the apparent quantum yield by photosynthesis (*AQY*) as well as an increase of 11.23% in the dark respiration rate (*Rd*) in tomato seedling leaves. In the absence of NaCl stress, the application of exogenous Se had no significant effects on the values of *AQY* and *Rd* in tomato seedling leaves but counteracted the effect of NaCl stress on *AQY* and Rd. This demonstrated that the application of exogenous Se can effectively enhance the utilization of optical energy by tomato seedling leaves under NaCl stress, resulting in the reduced degradation of photosynthates and their consequent accumulation. As shown in **Figures 6D, F**, the application of exogenous Se had no significant effects on the *Jmax* and *Vcmax* of tomato seedling leaves. NaCl stress led to a significant reduction in the *Jmax* and *Vc max* of tomato seedling leaves by 24.62% and 33.62%, respectively, while the application of exogenous Se led to an increase in the *Jmax* and *Vcmax* of tomato seedling leaves under NaCI stress. This suggests that the application of exogenous Se can relieve the inhibition of CO_2_ carboxylation and the regeneration of RuBP by salt stress.

### The activity of the key enzymes involved in the Benson-Calvin cycle

**Figure 7:**
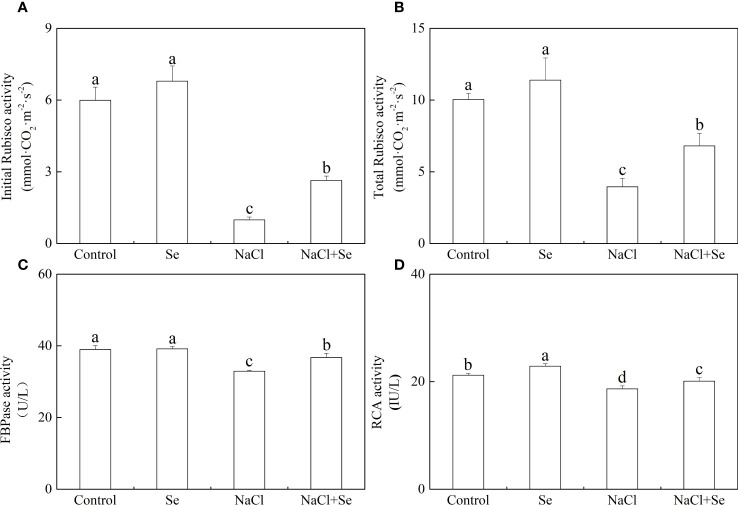
Effects of exogenous Se on the initial and total activity of ribulose-1,5-bisphosphate carboxylase (Rubisco) **(A, B)**, the activity of furetose-l,6-bisphosphate phosphatase (FBPase) **(C)** and Rubisco activating enzyme (RCA) **(D)** in leaves of tomato seedlings under NaCl stress. All the measurements were made on leaves at 9 d after treatment. Error bars represent SD (n=3). Different letters indicate the significance differences among the treatments (*P* < 0.05). Control: no Se and no NaCl; Se: 0.01 mM Na_2_SeO_3_; NaCl: 100 mM NaCl; and NaCl+Se: 0.01 mM Na_2_SeO_3_+100 mM NaCl.

As shown in **Figure 7**, the application of exogenous Se had negligible effects on the initial and overall activity of Rubisco and FBPase but led to an enhanced RCA activity. Salt stress led to a significant reduction in the initial and overall activity of Rubisco by 83.47% and 60.63%, respectively, reducing the activity of FBPase by 15.52% and that of RCA by 11.96%. However, the addition of exogenous Se alleviated the inhibitory effect of NaCl on these enzymes. In comparison with the NaCl group, the application of exogenous Se under salt stress improved the initial and overall activity of tomato seedling Rubisco, and the activities of FBPase and RCA by 166.18%, 72.11%, 11.57%, and 7.79% respectively.

### Level of transcription of the genes for the key enzymes involved in carbon assimilation

**Figure 8:**
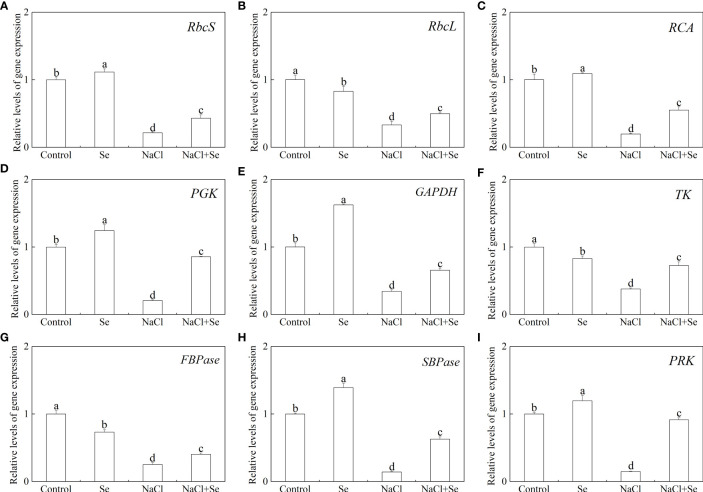
Effects of exogenous Se on the transcriptions of nine genes involved in the Calvin cycle in leaves of tomato seedlings under NaCl stress. RbcS (Rubisco small subunit gene, **A**); *RbcL* (Rubisco large subunit gene, **B**); *RCA* (Rubisco activase gene, **C**); *PGK* (3-Phosphogiyceric acid kinase gene, **D**); *GAPDH* (glyceraldehyde-3-phosphate dehydrogenase gene, **E**); *TK* (Transketolase gene, **F**); *FBPase* (Furetose-1,6-bisphosphate phosphatase gene, **G**); *SBPase* (Sedoheptulose-1,7-bisphosphatase gene, **H**); *PRK* (Ribulose-5-phosphate kinase gene, **I**). All the measurements were made on leaves at 9 d after treatment. Error bars represent SD (n = 3). Different letters indicate the significance differences among the treatments (*P < 0.05*). Control: no Se and no NaCl; Se: 0.01 mM Na_2_SeO_3_; NaCl: 100 mM NaCl; and NaCl+Se: 0.01 mM Na_2_SeO_3_+100 mM NaCl.

To further investigate the regulatory role of Se in photosynthesis, the levels of transcription of nine Calvin cycle genes, encoding Rubisco large subunit (RbcL), Rubisco small unit (RbcS), Rubisco activase (RCA), sedoheptulose-1,7-bisphosphatase (SBPase), phosphoglycerate kinase (PGK), phosphoribulokinase (PRK), thymidine kinase (TK), FBPase, and glycerol-3-phosphate dehydrogenase (GADPH), were investigated. As shown in **Figure 8**, the application of Se alone led to a significant upregulation in the levels of expression of *SBPase*, *PGK*, *PRK*, *GAPDH*, *RCA* and *RbcS* in tomato leaves, and significantly downregulated the levels of expression of *TK*, *FBPase* and *RbcL*. The NaCl stress led to a significant reduction in the levels of transcription of these genes. The application of exogenous Se led to a significant upregulation in the levels of transcription of nine key genes, associated with carbon assimilation in tomato seedlings under NaCl stress.

## Discussion

Biomass is one of the most reliable indicators that can directly reflect if plants are subject to adverse stress or exogenous substances and whether they can alleviate the effects of stress (Regni et al., 2021). The results indicated that salt stress led to a restriction in the growth of tomatoes, and the application of Se can effectively alleviate the inhibition of growth of tomato seedling by stress and improve the salt tolerance of tomato seedlings.

Ionic equilibrium reflects the stability of the internal environment of plant cells, which is one of the preconditions for the normal development of various physiological and biochemical processes in the cell (Thabet and Alqudah, 2023). Under salt stress, the excessive buildup of Na^+^ causes the outflow of K^+^ and Ca^2+^ from the cytosol, which upsets the balance of intracellular ions and interferes with plants’ regular metabolic processes (Ahanger and Agarwal, 2016; Cabot et al., 2014). Ca and Mg ions are involved in the regulation of plant growth through their influence on key metabolic pathways, including N assimilation, antioxidant metabolism, and the transduction of cell stress signals. These ions are also important inorganic ions for osmoregulation in plants. Therefore, reestablishing the balance of these ions in the cell is essential for plant growth under salt stress (Ahanger et al., 2015; Ahmad et al., 2016). It has been demonstrated that the application of exogenous nitrous oxide (NO) (Zhang et al., 2006), glutathione (GSH) (Zhou et al., 2019), putrescine (Shu et al., 2015), melatonin (Jiang et al., 2016), and silicon (Si) (Gengmao et al., 2015) reduces the contents of Na^+^ and Cl^–^ of the plant leaves or root system, increases the ratios of K^+^/Na^+^, K^+^/Ca^2+^and K^+^/Mg^2+^, and maintains ionic homeostasis, thus, enhancing the salt resistance of the plants. Drahonovsky et al. reported that spraying selenate on leaves could promote the absorption of K, Mg, Ca, S and Zn in different kinds of trees (Drahoňovský et al., 2016). Elkelish et al. found that exogenous Se facilitates the growth of wheat (*Triticum aestivum* L.) seedlings under salt stress, which resulted from the limited accumulation of toxic sodium ions in the upper leaves of plants and the maintenance of a high K/Na ratio (Elkelish et al., 2019). In this study, the application of exogenous Se under salt stress can effectively inhibit the accumulation of Na and increase the ratios of K/Na and Mg/Na, thus, maintaining the ionic homeostasis of root system and leaves of tomato seedlings and relieving the toxicity of Na. Additionally, this study found that salt stress had no significant effect on the selective absorption and transfer ability for Na and K, Ca and Mg from the roots to leaves, which is inconsistent with the research results of Zhou et al. (2019) that salt stress improved the selective absorption and transfer ability of Na and retarded the selective transfer of K, Ca, and Mg from the root system to the leaves in tomato seedlings. This could be owing to the different varieties of tomato. However, application of the exogenous Se also did not have a positive regulatory effect on the selective absorption and transfer ability of K, Ca, and Mg from the roots to leaves under salt stress (**Figure 3**).

The damage caused by salt does not only depend on ion concentration and ionic equilibrium but is also closely related to the micro-domain distribution equilibrium of salt in plant tissues. Under salt stress, the “salt spots” in the leaves and root systems of some plants are an indication of the non-uniform distribution of salt, which damages the plants. Recently, some studies indicated that the regulation of exogenous putrescine (put) on the uniformity of micro-domain ionic distribution in the root system was one of the mechanisms by which put enhanced the resistance of cucumber (*Cucumis sativu*s.) seedlings to salt stress (Yuan et al., 2019). The enhancement of the salt tolerance of tomato seedlings that followed the application of exogenous GSH also correlated with the improvement of uniformity of the micro-domain distribution of Na^+^, Cl^-^, and K^+^ in the leaves and root system (Zhou et al., 2019). In this study, X-ray energy spectrum analysis revealed that the relative contents of Na^+^ and Cl^-^ in all the tissues of leaves and root system of tomato seedlings increased significantly under salt stress, and Na^+^ and Cl^-^ were primarily concentrated in the cortex of root system and the xylem and phloem of the leaves. This was consistent with the findings of Zhou et al. (2019). Additionally, salt stress changed the distribution of K^+^, Ca^2+^, and Mg^2+^ in the leaves and root cells. K^+^ primarily accumulated in the phloem of leaves under non-stress conditions but was found to be concentrated in the xylem and sponge tissues under salt stress (**Tables 3**, **4**). These results indicated that the imbalance of micro-domain ionic distribution in the leaves and root cells and the accumulation of toxic ions, such as Na^+^ and Cl^–^, were the primary reasons for the damage of tomato seedlings under NaCl stress. The application of exogenous Se under NaCl stress led to a decrease in the relative contents of Na^+^ and Cl^-^ in all the tissues of tomato leaves and root system, increasing the relative contents of Na^+^ rendering them rather similar to those in the control group, and improved the microregional distribution uniformity of K^+^, Ca^2+^, and Mg^2+^ in the leaves and root system (**Tables 3**, **4**). These changes indicated that the application of exogenous Se alleviated the damage of salt stress to tomatoes and improved the salt tolerance of tomatoes by reducing the accumulation of salt ions in the root cells and improving the micro-domain ionic distribution in the leaves and root system under salt stress.

Under salt stress, the inhibition of plant growth is always accompanied by degraded photosynthetic capacity (Zhang et al., 2020). Under adverse stress, including salt stress, maintenance and restoration of photosynthetic function is one of the important mechanisms of plant resilience. The decrease in plant Pn was primarily influenced by stomatal and non-stomatal factors. In this study, the increase in Pn after the application of Se under salt stress was accompanied by an increase in the Gs, Ci, and Tr (**Figure 4**),which suggested that exogenous Se attenuated the stomatal limitations induced by osmotic stress under salt stress. This resulted in the ability of the leaf to contain high levels of CO_2_ and photosynthetic substrate, thus, alleviating the restriction of salt stress on Pn. This is consistent with the findings of previous studies by Khoshbakht et al. (2018). Additionally, exogenous Se also alleviated the negative effects of salt stress on *Vcmax*, *Jmax*, *AQY* and *Rd* (**Figure 6**). It was suggested that exogenous Se could also alleviate the reduction of Pn under salt stress by adjusting non-stomatal factors, such as the photochemical activity of PSII and the efficiency of carbon assimilation.

The chloroplast and PSII reaction center on the thylakoid membrane structure are the primary sites where light energy is converted and utilized in plants. It is the primary site of damage to the plant photosynthetic apparatus caused by adverse stress, which includes salt stress. Excess light energy induced by adverse stress can cause photooxidative damage to PSII and decreases the activity of the PSII reaction center. This results in severe photo inhibition. To prevent and alleviate photoinhibition, plants have developed a series of photoprotective mechanisms during their long evolutionary process (Pinnola and Bassi, 2018; Quaas et al., 2015). Our previous work has demonstrated that salt stress damaged PSII in the tomato seedling leaves and subsequently caused photoinhibition. The addition of exogenous Se protected PSII from damage from surplus energy and improved the activity of PSII by initiating the ROS scavenging activity and the NPQ mechanism in the chloroplast (Diao et al., 2014). However, the regulatory role of exogenous Se on the distribution of activation energy between photosystems under salt stress remains unclear. State transitions regulation is a quick response photoprotective mechanism for photosynthetic organisms to regulate the balance of activation energy that is distributed between the two photosystems and reduce the activation energy pressure on the PSII reaction centers (Rochaix, 2014). Under adverse stress, maintaining the balanced distribution of activation energy is the prerequisite for the efficient operation of PSII and the coordination of the linear electron transfer chain (LETC). In this study, salt stress resulted in an imbalance of the distribution of activation energy between PSI and PSII and excess light energy (**Figure 5**), which could be an important reason for the damage to PSII under salt stress. Under salt stress, the application of exogenous Se led to a significant increase in the *P* value and a decrease in *β/α-1*, *D* and *Ex*, thus implying that the application of exogenous Se under salt stress reduced the photooxidation damage and increased the light energy utilization efficiency of PSII. This effect takes place by the initiation of the state transitions control mechanism, which reduced the imbalance of activation energy distributed between PSI and PSII. Thus, the share of activation energy used for photochemical reaction processes increased, which resulted in a decrease in the excess light energy.

**Figure 9:**
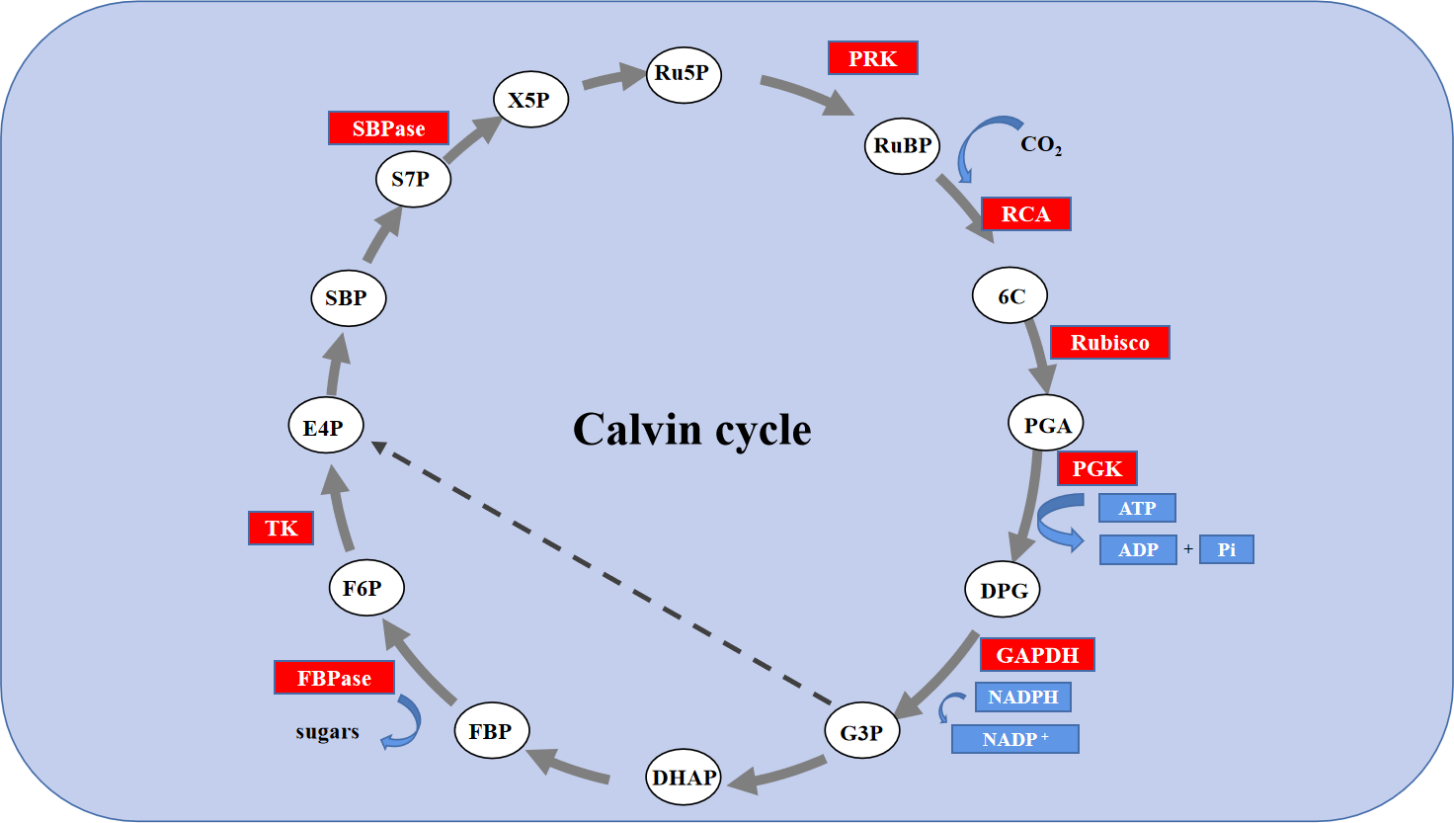
Schematic diagram of the expression of Calvin cycle-related gene expression in leaves of tomato seedlings under salt stress with Se application. The red background color represents the up-regulation of genes; Enzymes: RCA, Rubisco activating enzyme; Rubisco, Ribulosebisphosphate carboxylase/oxygenase; PGK, 3-phosphoglycerate kinase; GAPDH, glyceraldehyde-3-phosphate dehydrogenase; FBPase, Fructose 1,6-bisphosphatase; TK, transketolase; SBPase, sedoheptulose 1,7-bisphosphatase; PRK, phosphoribulokinase. Metabolites: RUBP, ribulose-1,5-bisphosphate; 6C, An unstable 6-carbon compound; PGA, 3-phosphoglycerate; DPG, 1,3-bisphosphoglycerate; G3P, glyceraldehyde-3-phosphate; DHAP, dihydroxyacetone phosphate; FBP, fructose-1,6-bisphosphate; F6P, fructose-6-phosphate; E4P, erythrose-4-phosphate; SBP, sedoheptulose-1,7-bisphosphate; S7P, sedoheptulose-7-phosphate; X5P, xylulose-5-phosphate; RU5P, ribulose-5-phosphate; sugars, Glucose-6-phosphate.

The Calvin cycle is the primary route to fixation of CO_2_ in C3 plants. The Calvin cycle consists of three stages, including carboxylation, carbon reduction, and the regeneration of RuBP (Tang et al., 2018). It has been reported that the efficiency of Rubisco carboxylation and the activities of key genes related to the Calvin cycle are highly significant for enhancing photosynthesis under abiotic stress (Fatma et al., 2016; Feng et al., 2021). Feng reported that melatonin improved the activity of key enzymes in the Calvin cycle and the corresponding degree of expression of mRNA in the leaves of cucumber (*Cucumis sativus* L.) seedlings to enhance the efficiency of their assimilation of photosynthetic carbon, thereby, improving their tolerance to cold (Feng et al., 2021). However, the role of exogenous Se in the Calvin cycle and the corresponding process remains unclear. Thus, in this study, the use of an A/Ci curve and the activity of key enzymes Rubisco, RCA, and FBPase of the Calvin cycle and transcription of eight genes proved that owing to enzymatic activity and the positive regulation of its expression, Se could regulate the photosynthetic capacity. Rubisco is the rate-limiting enzyme in photosynthetic carbon assimilation. It catalyzes the first step in CO_2_ fixation to convert RuBP and CO_2_ to two molecules of 3-phosphoglycerate (PGA). Thus, this enzyme activity directly affects the rate of CO_2_ assimilation (Darabi and Seddigh, 2017; Masumoto et al., 2015). *RbcS* and *RbcL* encode the large and small subunits of Rubisco, respectively, which work closely to regulate the structure and function of the Rubisco holoenzyme. RCA enables Rubisco to be maximally activated under *in vivo* conditions, and its activity influences the carboxylation efficiency of Rubisco. Thus, salt stress led to a significant degradation in the initial and overall activity of Rubisco and that of RCA, the decrease in *Vc max*, and the decreased levels of expression of *RbcL*, *RbcS* and *RCA*. However, exogenous Se improved the activity of these enzymes and the levels of expression of these genes under salt stress. Therefore, the application of exogenous Se plays an active role in enhancing the efficiency of CO_2_ carboxylation and photosynthetic electron transfer efficiency, promoting the formation of NADPH and ATP by increasing the activity of RCA and activating the state of Rubisco in leaves of salt-stressed tomato seedlings. The regeneration of RuBP requires the use of ATP and NADPH, which are generated by photoreaction. Thus, the regeneration of RuBP is not only involved in the electron transport chain but also the enzymes downstream of Rubisco in the Calvin cycle. FBPase is the key enzyme involved in the regeneration of RuBP, which irreversibly catalyzes the first step in the conversion of propyl phosphate to sucrose (Wu, 2013). TK, SBPase, and PRK are also involved in the regeneration of RuBP. Among of them, SBPase controls the influx and regeneration of carbon during the Calvin cycle. PRK directly catalyzes the regeneration of RuBP, which is the receptor for CO_2_ fixation. In this study, exogenous Se alleviated the activity of FBPase and the expression of genes induced by salt stress to various degrees, as well as the levels of expression of *TK*, *SBPase*, and *PRK*. In addition, it increased *Jmax*, indicating that the application of exogenous Se could promote the regeneration of RuBP in the Calvin cycle, thereby enhancing the efficiency of Rubisco carboxylation of the tomato seedling leaves under salt stress (**Figure 6D, F**, **7**). Additionally, exogenous Se also led to a significant upregulation in the levels of expression of *PGK* and *GAPDH* under salt stress. PGK and GAPDH are enzymes involved in carbon reduction and directly affects the efficiency of transport of photosynthetic carbon assimilation, suggesting that application of exogenous Se under salt stress could promote the transport of Calvin cycle and alleviate the feedback inhibition of photosynthate, thus, improving the photosynthetic efficiency (Kubo et al., 2016). Therefore, the application of Se to leaves under salt stress improved the key enzyme activity of the Calvin cycle and the expression of genes in tomato leaves, thereby alleviating the inhibition of salt stress on photosynthesis by tomato seedlings (**Figure 9**). This could be an important mechanism for Se to improve the photosynthetic performance of tomato seedlings and promote their growth.

## Conclusion

The application of 0.01 mmol•L^-1^ exogenous Se under salt stress effectively blocked the leaves and root system of tomato seedlings from absorbing Na, promoted the absorptions of K, Ca, and Mg to varying degrees, and improved the micro-domain ionic distribution in the leaves and root system under salt stress, thus, relieving the imbalance in ionic homeostasis and ion toxicity induced by salt stress. Moreover, the exogenous Se overcame the stomatal limitation, initiated the state transitions mechanism between PSI and II, and up-regulated the initial and overall activity of Rubisco, RCA and FBPase, and the expression levels of nine genes. Thus, the application of exogenous Se improves the concentration of photosynthetic substrates, ensures a balance of activation energy distribution between PSI and II, and promotes the carboxylation efficiency of CO_2_ and carbon assimilation, which improves the photosynthetic efficiency of tomato seedling leaves under salt stress. Therefore, the results suggested that a supply of Se can alleviate the inhibition of salt stress on tomato seedling growth by rebuilding ionic homeostasis and promoting photosynthetic capacity.

## Data availability statement

The original contributions presented in the study are included in the article/supplementary files. Further inquiries can be directed to the corresponding authors.

## Author contributions

This work was carried out in collaboration between all the authors. MD and H-yL defined the research theme and designed the experiment. WZ and XH performed the experiments and wrote the manuscript. XC analyzed the data, interpreted the results, and prepared the figures. HH and BS modified the manuscript. All authors contributed to the article and approved the submitted version.
